# Mobilization and Role of Starch, Protein, and Fat Reserves during Seed Germination of Six Wild Grassland Species

**DOI:** 10.3389/fpls.2018.00234

**Published:** 2018-02-27

**Authors:** Ming Zhao, Hongxiang Zhang, Hong Yan, Lu Qiu, Carol C. Baskin

**Affiliations:** ^1^Jilin Provincial Key Laboratory of Grassland Farming, Northeast Institute of Geography and Agroecology, Chinese Academy of Sciences, Changchun, China; ^2^College of Life Sciences, Northeast Normal University, Changchun, China; ^3^Department of Biology, University of Kentucky, Lexington, KY, United States; ^4^Department of Plant and Soil Sciences, University of Kentucky, Lexington, KY, United States

**Keywords:** fat, germination, protein, seed reserve mobilization, soluble sugar, starch, wild species

## Abstract

Since seed reserves can influence seed germination, the quantitative and qualitative differences in seed reserves may relate to the germination characteristics of species. The purpose of our study was to evaluate the correlation between germination and seed reserves, as well as their mobilization during germination of six grassland species (*Chloris virgata*, *Kochia scoparia*, *Lespedeza hedysaroides*, *Astragalus adsurgens*, *Leonurus artemisia*, and *Dracocephalum moldavica*) and compare the results with domesticated species. We measured starch, protein, and fat content in dry seeds and the initial absorption of water during imbibition. Starch, soluble protein, fat, and soluble sugar content also were determined at five stages during germination. Starch, protein, and fat reserves in dry seeds were not significantly correlated with germination percentage and rate (speed), but soluble sugar and soluble protein contents at different germination stages were positively significantly correlated with germination rate for the six species. Starch was mainly used during seed imbibition, and soluble protein was used from the imbibition stage to the highest germination stage. Fat content for all species remained relatively constant throughout germination for six species, regardless of the proportion of other seed reserves in the seeds. Our results for fat utilization differ from those obtained for cultivated grasses and legumes. These results provide new insight on the role of seed reserves as energy resources in germination for wild species.

## Introduction

Seed germination is the beginning of the life history for seed plants (Donohue et al., 2010; Nonogaki et al., 2010; Lai et al., 2015). It has been suggested that one way in which the environment indirectly affects seed germination is through the types and amounts of compounds transferred from the mother plant to the seeds (Donohue, 2009; Baskin and Baskin, 2014; Li et al., 2017). This transfer of compounds to seeds includes carbohydrates, proteins and lipids, which are the major reserves in most seeds (Alencar et al., 2012).

The definition of germination *sensu stricto* is the complex process from water uptake of dry seeds (imbibition) to radicle protrusion through the seed coat (Weitbrecht et al., 2011; Rajjou et al., 2012). With regard to germination, there are two fundamental seed biology questions. What metabolic activities are involved, and how are energy reserves used to support embryo growth and radicle protrusion (Rosental et al., 2014)? Although seed reserve mobilization is usually thought to be a post-germination process (Eastmond and Graham, 2001; Pritchard et al., 2002), some studies have found that seed reserve mobilization or degradation of starch (Galland et al., 2017), protein (Yang et al., 2007; Rosental et al., 2014), and lipid (Sreenivasulu et al., 2008) occurs during germination. Also, storage protein (e.g., 11S globulins) mobilization can occur during the seed maturation process, albeit to a lower extent than during seed germination and seedling establishment (Job et al., 1997; Bourgne et al., 2000; Gallardo et al., 2001).

It has been reported that seed reserve content is correlated with germination percentages and/or rates (speed) (Soriano et al., 2014). For example, the starch content in *Citrullus lanatus* seeds was positively correlated with germination rate but not with the final germination percentage (Wang et al., 2011). Soluble sugar (such as glucose and sucrose) was positively correlated with germination percentage of *Medicago truncatula* seeds (Vandecasteele et al., 2011). Protein content was positively correlated with germination percentage of *Pinus pinaster* (Wahid and Bounoua, 2013) and the germination rate of *Solanum tuberosum* (Bhatt et al., 1989) seeds. No significant correlation was found between fat content and germination percentage of *Linum usitatissimum* seeds (Kanmaz and Ova, 2015), but fatty acid content was negatively correlated with germination percentage of *Gossypium* spp. seeds (Hoffpauir et al., 1950). Thus, the influence of seed reserves on germination depends on the amount of reserve and the plant species. However, little information is available about the effect of the combination of reserves on germination traits at the interspecific level (Soriano et al., 2011). Different seed reserves may have different roles during germination for various species (Gu et al., 2016). For example, oil is utilized during germination of *Sorghum bicolor* seeds (Elmaki et al., 1999), but it is not essential for germination of *Arabidopsis thaliana* seeds (Pritchard et al., 2002). Therefore, we hypothesized that neither starch, protein nor fat content of dry seeds may be significantly correlated to germination percentages and rates at the interspecific level.

The mobilization of seed reserves during germination varies with the amount of reserves and the species. Starch was present in the highest amount, and its degradation was highest among the various seed reserves used during germination of *Oryza sativa* (Palmiano and Juliano, 1972), *Sorghum bicolor* (Elmaki et al., 1999), and *Avena sativa* (Xu et al., 2011) seeds. Protein was mainly mobilized and used during germination of the legume species *Dalbergia nigra* (Ataíde et al., 2013). Seeds of *Helianthus annuus* (Erbaş et al., 2016) and *Sterculia urens* (Satyanarayana et al., 2011) have high protein and oil content, which decreased dramatically during germination of these two species. Therefore, we hypothesized that the seed reserve present in the highest amount is the one that is most heavily utilized during germination. Additionally, the various metabolic processes that occur during germination *sensu stricto* start at different times for individual seeds. Consequently, it is difficult to determine the boundary between germination and seedling growth for a seed population, as seeds do not complete the germination process synchronously (Gallardo et al., 2001). In this context, it is worth noting that seeds are known to be extremely heterogeneous in biochemical terms (Still and Bradford, 1997; Bourgne et al., 2000). Thus, in our study we divided the seed germination process of six species into different stages, including imbibition, 1% germination, 50% germination, highest germination and finally the seedling stage to investigate seed reserve mobilization.

The relationship between seed reserves and germination has been studied mainly in economically-important species such as *Oryza sativa* (Sun et al., 2015), *Glycine max* (Bellaloui et al., 2017), *Sorghum bicolor* (Elmaki et al., 1999), *Linum usitatissimum* (Kanmaz and Ova, 2015) and in the model plant species *Medicago truncatula* (Vandecasteele et al., 2011) and *Arabidopsis thaliana* (Shrestha et al., 2016). These previous studies on seed reserve mobilization during germination have given little attention to wild grassland species and have mainly focused either on only one type of reserves (Kim et al., 2009; Shaik et al., 2014) or a single species (usually crops) (Goyoaga et al., 2011; Ataíde et al., 2013; Lopes et al., 2013).

In our study, we selected six wild species from the Songnen grassland in northeast China to test our hypotheses. These species were selected because the most abundant storage reserve in the seeds was starch, protein, or lipid, and their germination percentages were high, allowing us to collect samples during different germination stages for the seed populations. Specifically, we asked: (1) Does the type and amount of seed reserve influence germination? (2) Is the most abundant reserve compound the most heavily used one during germination? Also, we compared the results for these grassland species with those for cultivated species. Such comparisons would provide some new insights on seed physiology of wild species and on changes that may have occurred in seed reserve utilization/physiology during the domestication of crop species.

## Materials and Methods

### Study Species, Seed Collection, and Storage

The six wild species selected for study include *Chloris virgata*, *Kochia scoparia*, *Lespedeza hedysaroides*, *Astragalus adsurgens*, *Leonurus artemisia*, and *Dracocephalum moldavica*. These species occur in four families and four of them are annuals and two are perennials (**Table 1**). Seeds of *C. virgata* have endosperm but those of the other five species do not have endosperm. Seeds of each species were collected during autumn 2015 from wild populations on the Songnen plain (123° 44–47′ E, 44° 40–45′ N) situated in northeast China. In this region, the average annual temperature and precipitation are around 5°C and 400 mm (calculated from the recent 30 years), respectively (Dong et al., 2011). Seeds were dry-stored in cloth bags at room temperature until the start of the experiment in May 2016. One hundred seeds were selected randomly and weighed with an electronic balance (0.1 mg, METTLER TOLEDO ME204), with five replicates for each species. Thousand seed weights were calculated.

**Table 1:** Family, life cycle, photosynthetic pathway (P), main seed reserve proportion in dry seeds and 1000 seed mass of the six species in this study.

Species	Family	Life cycle	P	Starch (%)	Protein (%)	Fat (%)	Seed mass (g)
*Chloris virgata*	Poaceae	Annual	C_4_	62.2	19.9	6.2	0.5461
*Kochia scoparia*	Amaranthaceae	Annual	C_4_	47.3	24.2	11.2	0.4544
*Lespedeza hedysaroides*	Fabaceae	Perennial	C_3_	48.2	23.3	8.6	2.0691
*Astragalus adsurgens*	Fabaceae	Perennial	C_3_	47.8	23.8	9.4	1.4723
*Leonurus artemisia*	Lamiaceae	Annual	C_3_	35.2	20.2	35.8	1.2388
*Dracocephalum moldavica*	Lamiaceae	Annual	C_3_	25.6	17.0	20.0	1.8680


### Water Uptake during Imbibition

Seeds were incubated on the surface of two layers of filter paper moistened with distilled water in 10 cm diameter Petri dishes. Four replicates of 25 seeds each were used for each species. Seeds were incubated at a constant temperature of 25°C, with a 12-h daily photoperiod (hereafter light) (Sylvania cool white fluorescent lamps, 25 μmol photons m^-2^ s^-1^, PAR). The rate of water absorption was determined for each species, and the experiment was terminated when the first seed(s) with an emerged radicle was(were) observed. According to a preliminary experiment, germination started between 2 and 42 h after seeds were placed on wet filter paper, depending on the species. During the imbibition period, total seed mass in each Petri dish was measured 5–6 times from the beginning of incubation (dry seeds) with the same time interval for each species (Supplementary Table S1). Surface water was removed from seeds with filter paper each time they were weighed. The seeds were put back into the dishes after they were weighed. Water uptake (%) was calculated as (W_2_–W_1_) × 100/W_1_. W_1_ and W_2_ represent the mass of seeds at time zero and at various sampling times during imbibition, respectively.

### Germination Characteristics

Seeds were immersed in 70% (v/v) ethanol for 1 min for surface sterilization and then washed three times with distilled water. Seeds were incubated on the surface of two layers of filter paper moistened with distilled water in 10 cm diameter Petri dishes, and there were three replicates of 50 seeds each for each species. Distilled water was added when necessary to keep the filter paper moist. The experiment was conducted in light at 25°C. Except for the two fast germinating species, *C. virgata* and *K. scoparia*, which were checked at 2 h intervals, germination was recorded every 12 h. Seeds were considered to be germinated when the radicle emerged. The experiment lasted for a total of 7 days, at which time germination was completed. The germination percentage (number of germinated seeds/total seeds) and rate or speed (the reciprocal of time to 50% germination in days) were calculated.

### Dynamics of Seed Reserves during Germination

Total starch, protein, and fat content were measured in dry seeds of the six species. To analyze starch content, samples of dry seeds were ground and extracted twice with 80% ethanol and then twice with 52% perchloric acid. Starch content was determined with the anthrone-sulfuric acid method (Fernandes et al., 2012) at 640 nm with a Spectrometer (723 UV-VIS, Shandong, China). Samples of dry seeds were ground and extracted with 98% sulfuric acid. Total nitrogen content of dry seeds was measured with a Continuous Flowing Analyzer (Kjeltec 8400, Germany) and then total protein content was calculated by multiplying nitrogen content by the constant 6.25 (Soriano et al., 2011). To analyze fat content, dry samples of seeds were ground and ethylic-ether was used as extraction buffer. Concentration of fat was determined by the Soxhlet extraction method (Soriano et al., 2011) using Solvent Extractor SER (VELP Scientifica, Italy). The content of starch, protein, and fat (mg/g) in dry seeds was also calculated as a percent of seed mass for the six species (**Table 1**).

For measuring reserve mobilization during germination of the six species, 0.5 g seeds were weighed and put on moist filter paper in each of 15 Petri dishes for each species and incubated in light at 25°C. Three dishes (biological replicates) of seeds were taken for each species at each of five stages (Supplementary Table S2): imbibition (middle time from incubation to the start of germination, stage 1), start of germination (1% germination, stage 2), 50% germination (stage 3), highest germination (stage 4) and early seedling stage (around 80% with visible cotyledon (s), stage 5). Among the stages, only samples from stage 5 can be treated as seedlings with both shoot and root. Seeds from other stages were imbibed and/or had an emerged radicle. The experiment was performed three times. The first, second and third times the experiment was done, starch and soluble sugar, soluble protein, and fat were analyzed, respectively. The methods of starch and fat measurements during germination were the same as those described above for dry seeds. To analyze the protein fraction that can be used for germination, we measured the content of water-soluble protein (hereafter soluble protein). This part of soluble protein that we extracted could be surmised to be albumin fraction (Osborne, 1924). It is treated as one of the seed reserves in our study, but it may contain other substances, e.g., enzymes. One hundred milligrams of fresh seeds/seedlings were manually ground and extracted with distilled water. After centrifugation, soluble protein content was measured using the Coomassie Brilliant Blue (mixture of Coomassie Brilliant Blue G250, 90% ethanol, 85% phosphoric acid, and distilled water) method (Bradford, 1976) and determined at 595 nm with a Spectrometer (723 UV-VIS, Shandong, China). To analyze water-soluble sugar (hereafter soluble sugar) dynamics, seeds/seedlings were dried at 50°C for 72 h and manually ground. Distilled water was added to the samples and warmed at 80°C for 1 h. After centrifugation, soluble sugar content was determined with the anthrone-sulfuric acid method (Yemm and Willis, 1954) at 625 nm with a Spectrometer (723 UV-VIS, Shandong, China). To compare the changes of each seed reserve during germination at the same scale, the concentration (or content, mg/g) of starch, soluble protein, fat, and soluble sugar at each stage from imbibition (stage 1) to early seedling (stage 5) relative to content present at the imbibition stage was calculated as (C_2_–C_1_) × 100/C_1_, C_1_ and C_2_ represent the content (mg/g) at imbibition stage (stage 1) and content (mg/g) at the four subsequent sampling stages (stages 2–5) during germination and early seedling growth, respectively.

### Statistical Analysis

Germination data were transformed (arcsine) before statistical analysis was conducted to ensure homogeneity of variance. The correlations between starch, protein, and fat contents in dry seeds, soluble protein and soluble sugar at different germination stages, and germination characters (percentage and rate) were tested by Pearson correlation analysis. Regression analysis was used to fit the relationship between seed reserves and germination stages. One-way ANOVA was used to analyze the effect of species on germination rate and the effect of germination stages on seed reserve contents. Multiple comparison tests were used to compare differences among treatment means at 0.05 level. Statistical analyses were carried out in SPSS (version 18.0, SPSS, Inc., Chicago, IL, United States).

## Results

### Water Uptake during Imbibition

**FIGURE 1:**
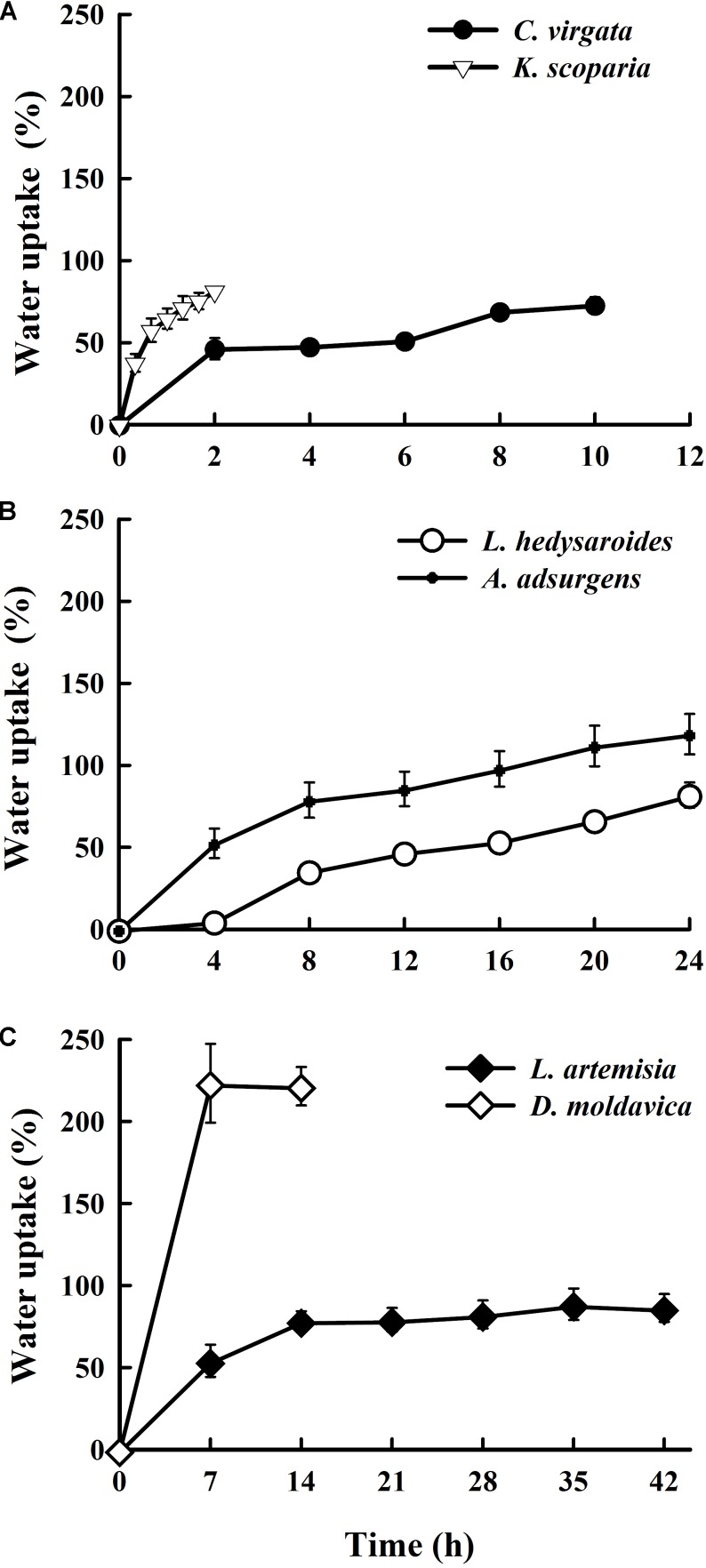
Water uptake (%) of *Chloris virgata*, *Kochia scoparia* **(A)**, *Lespedeza hedysaroides*, *Astragalus adsurgens* **(B)**, *Leonurus artemisia*, and *Dracocephalum moldavica* **(C)** seeds during imbibition at 25°C.

Seeds of *K. scoparia* and *C. virgata* quickly absorbed water during the first 20 min and 2 h of incubation, respectively, with the percentage of water absorbed approaching or exceeding half of the final amount (**Figure 1**). Imbibition of *C. virgata* and *K. scoparia* seeds reached 73 and 81.6% after 10 and 2 h, respectively. For *L. hedysaroides* and *A. adsurgens*, water was rapidly imbibed during the first 8 h, after which seeds continued to absorb water. The quick water uptake stage for *L. artemisia* and *D. moldavica* was 7 h, after which water content reached 54 and 223%, respectively. We stopped measuring water absorption by *D. moldavica* seeds after 14 h, because seeds became covered with mucilage that made it impossible to accurately determine the amount of water imbibed by the seeds. Except for *D. moldavica* with a 223% increase, the highest water uptake during the imbibition of the studied species ranged from 73 to 117%.

### Germination Percentage and Rate

**FIGURE 2:**
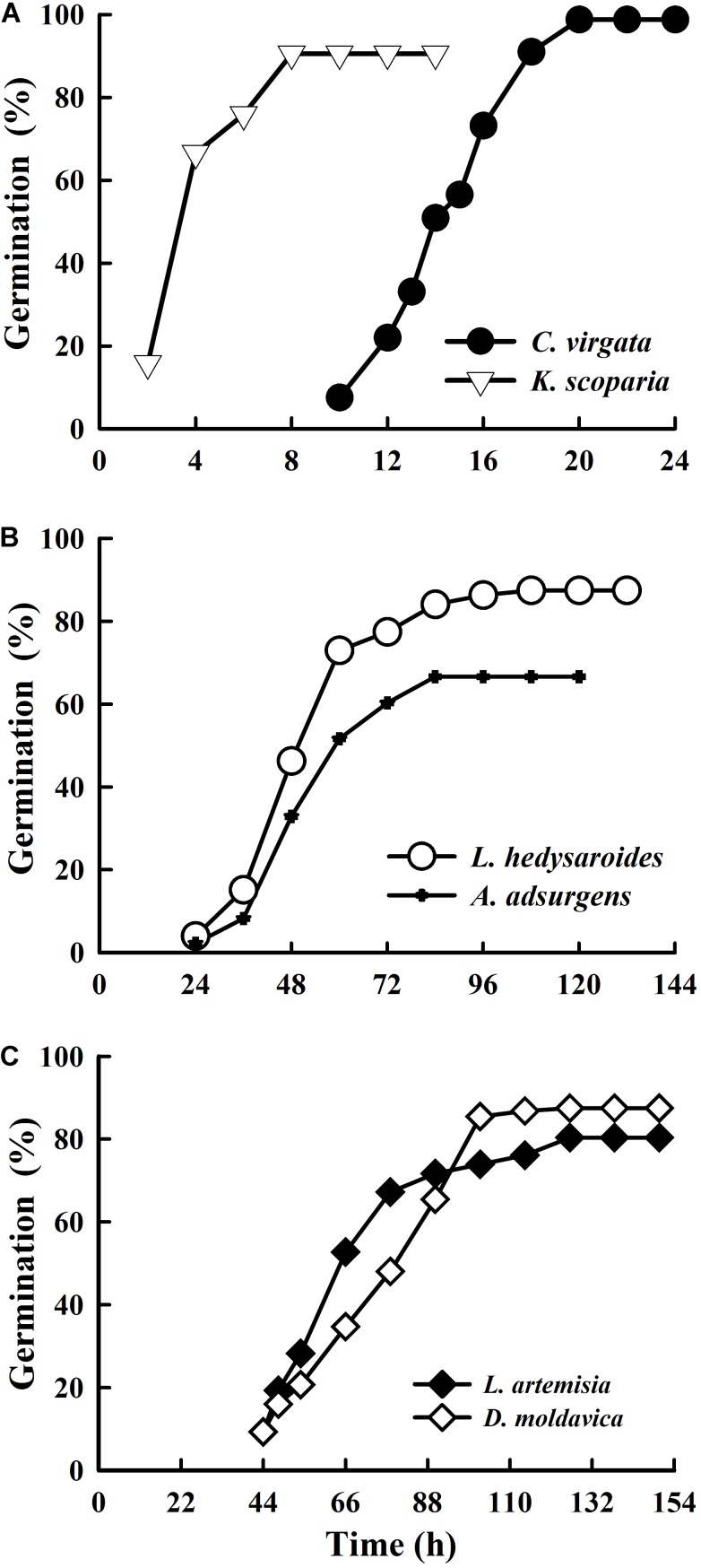
Cumulative seed germination percentages of *Chloris virgata*, *Kochia scoparia* **(A)**, *Lespedeza hedysaroides*, *Astragalus adsurgens* **(B)**, *Leonurus artemisia* and *Dracocephalum moldavica* **(C)** seeds in light at 25°C.

Germination of the five species was ≥80% (**Figure 2**), with that of *A. adsurgens* 67%. Seeds of *K. scoparia* and *C. virgata* started to germinate after 2 and 10 h, respectively, and reached the highest germination percentage within 8 and 20 h, respectively. Seeds of *L. hedysaroides* and *A. adsurgens* began to germinate after 24 h and achieved the maximum after 108 and 84 h, respectively. While seeds of *L. artemisia* and *D. moldavica* started to germinate after 44 h and gradually reached the highest germination after 126 h.

**FIGURE 3:**
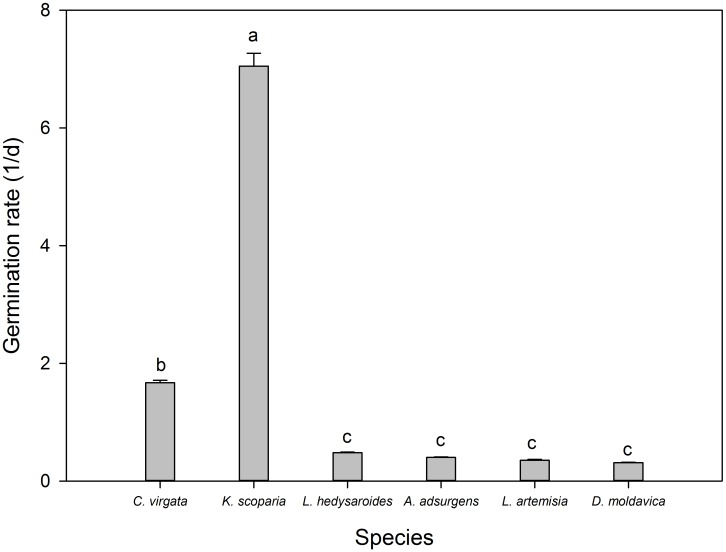
Germination rate of the six species *Kochia scoparia*, *Chloris virgata*, *Astragalus adsurgens*, *Lespedeza hedysaroides*, *Leonurus artemisia*, and *Dracocephalum moldavica*. Rates with different lowercase letters differed significantly at 0.05 level.

The germination rate of *K. scoparia* seeds was significantly higher than that of *C. virgata* seeds (**Figure 3**), which was 7.05 and 1.67, respectively, both significantly higher than that of the other four species (*P* < 0.05). Germination rates of the other species were not significantly different from each other, which ranged from 0.31 to 0.48 (*P* < 0.05).

### The Relationship between Seed Reserve Content and Germination

Dry seeds of *C. virgata* had a relatively high starch content (62.2%), and those of *K. scoparia*, *A. adsurgens*, and *L. hedysaroides* had high levels of starch (47.3–48.2%) and protein (23.3–24.2%) (**Table 1**). Seeds of *L. artemisia* had a relatively high fat and starch content (35.8 and 35.2%, respectively). Seeds of *D. moldavica* had more starch (25.6%) than protein (17.0%) or fat (20.0%), but the percentage of fat was higher than that of the non-Lamiaceae species. Germination percentages and rates were not correlated with the contents of the three major reserves in dry seeds of the six species, but germination rate was significantly correlated with soluble sugar and soluble protein contents at different germination stages for all species (*P* < 0.05, **Table 2** and Supplementary Table S4). Soluble sugar content was positively correlated with the starch content and negatively correlated with the fat content in dry seeds (*P* < 0.01). Soluble protein content was positively correlated with the protein content in dry seeds (*P* < 0.01). Additionally, the 1000 seed weight of the six species varied from 0.45 to 2.07 g (**Table 1**), and germination rate had a significant negative correlation with 1000 seed weight for all species (*P* < 0.01) (**Table 2**).

**Table 2:** Correlation coefficients between germination and seed reserve contents.

Source of variation	Starch^§^	Protein^§^	Fat^§^	Seed size	Soluble sugar^†^	Soluble protein^†^
GP	- 0.23	-0.09	0.02	-0.01	-0.02	0.30
GR	0.20	0.45	-0.27	-0.71**	0.48*	0.77**
Soluble sugar	0.64 **	0.22	-0.62**			
Soluble protein	0.16	0.67**	-0.40


*GP, germination percentage; GR, germination rate. ^§^The values used in the analysis were contents in dry seeds. ^†^The values used in the analysis were measurements at highest germination (stage 4), correlations with values at other stages was given in Supplementary Table S4. ^∗^Denotes significant at 0.05 level. ^∗∗^Denotes significant at 0.01 level.*

### Dynamics of Seed Reserves during Germination

**FIGURE 4:**
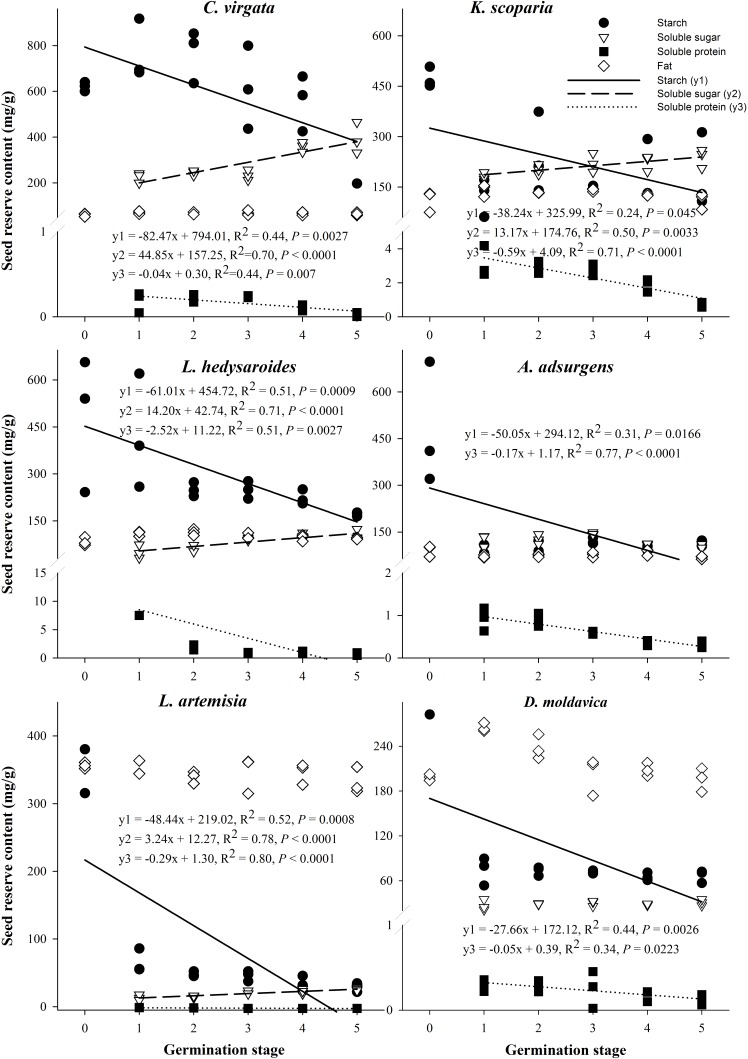
Regression analysis between starch, fat, soluble protein, soluble sugar, and germination stage for the six species *Kochia scoparia*, *Chloris virgata*, *Astragalus adsurgens*, *Lespedeza hedysaroides*, *Leonurus artemisia*, and *Dracocephalum moldavica*. (0) Dry seeds; (1) imbibition; (2) 1% germination; (3) 50% germination; (4) highest germination; (5) early seedling.

The starch and soluble protein content of the six species significantly decreased with the germination stage (*P* < 0.05, **Figure 4**), but fat content of the six species did not significantly change with germination stage (*P* > 0.05). The soluble sugar content of four of the six species significantly increased with germination stage (*P* < 0.05), with that of *A. adsurgens* increasing and then decreasing and that of *D. moldavica* remaining unchanged.

**FIGURE 5:**
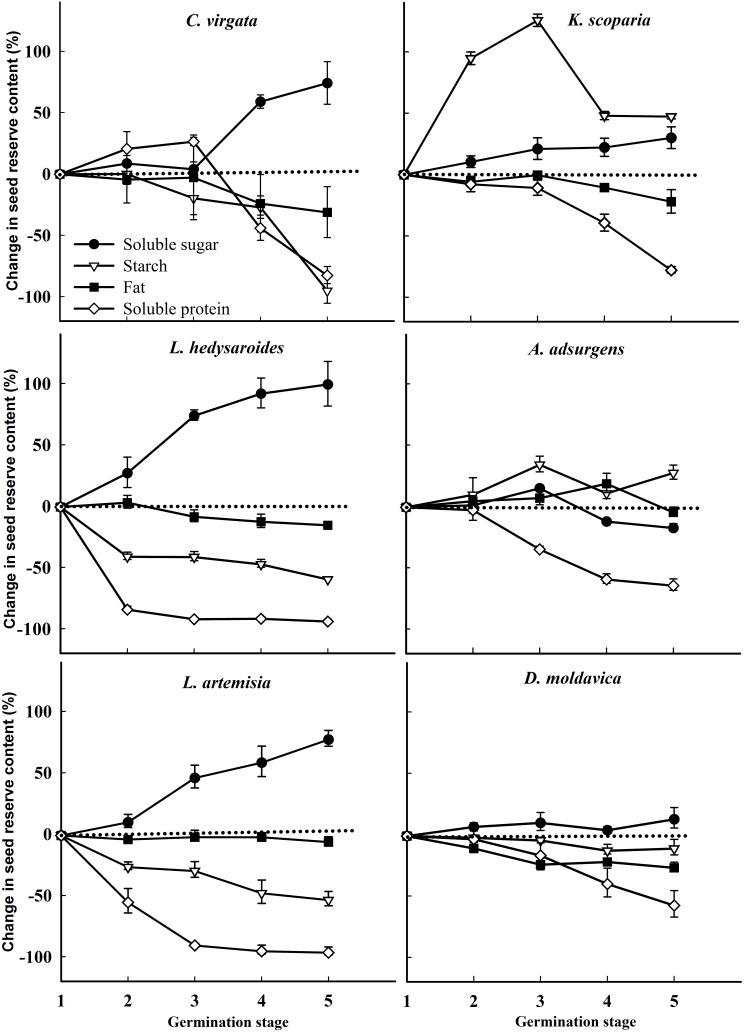
Change (%) in seed reserve content (mg/g) for *Chloris virgata*, *Kochia scoparia*, *Lespedeza hedysaroides*, *Astragalus adsurgens*, *Leonurus artemisia*, and *Dracocephalum moldavica* at various germination stages (relative to imbibed seeds). (1) Imbibition; (2) 1% germination; (3) 50% germination; (4) highest germination; (5) early seedling. The change in seed reserves was calculated from data in Supplementary Table S3.

For all species except *C. virgata* and *L. hedysaroides*, there was a significant decrease in starch when imbibed seeds were compared to dry seeds, and this was the main period of starch utilization (**Figure 4** and Supplementary Table S3). The main period of starch utilization for *L. hedysaroides* was during the imbibition (stage 1) to 1% germination (stage 2) (**Figure 5**), but the decrease in starch was not significant (*P* < 0.05). For *C. virgata*, starch content decreased significantly during the highest germination (stage 4) to early seedling stage (stage 5). The decrease in starch content ranged from 10.1 to 89.7% during the period from dry seeds to highest germination (stage 4) for the six species. Soluble protein content decreased to a larger proportion than the other two reserves from imbibition (stage 1) to the early seedling stage (stage 5) for the six species (**Figure 5**). However, a significant decrease occurred at different germination stages and the decrease ranged from 39.1 to 93.9% during imbibition (stage 1) to highest germination (stage 4) for the six species.

## Discussion

Water absorption during germination is related to the structure of the testa (Debeaujon et al., 2000; Dang et al., 2014) and the chemical composition of seeds (Brancalion et al., 2008). The two legume species *A. adsurgens* and *L. hedysaroides* have water-impermeable seed coats (physical dormancy), but the seeds imbibed water. Thus, the water gap (an anatomical structure in a water-impermeable seed coat that opens thereby permitting water entry into the seed, e.g., the lens on a legume seed) must have opened during storage (Baskin, 2003). However, the water gap is small, which would help explain the relatively slow rate of imbibition (**Figure 1**). Compared with the high-starch seeds (*C. virgata*) with a relatively low final percentage of water uptake, the high-protein seeds (*K. scoparia*, *L. hedysaroides*, and *A. adsurgens*) had a higher water absorption percentage at the end of imbibition, as protein contains more hydrophilic radicals than starch (Brancalion et al., 2008). Also, seeds with high protein content are known to imbibe more water than fatty seeds (Benech-Arnold and Sanchez, 2004), but it was not always the case in our study. Seeds of *L. artemisia* (20.2% protein) imbibed a similar amount of water (around 80%) as the two high-protein species *K. scoparia* (24.2% protein) and *L. hedysaroides* (23.3% protein), but less water than the seeds of *A. adsurgens* with 23.8% protein. For fat-storing *D. moldavica* seeds, water uptake reached 223%. This is likely related to the mucilage of the *D. moldavica* seed coat (Huang and Gutterman, 2004). Mucilage is a kind of pectinaceous polysaccharide, which can increase water uptake by seeds (Yang et al., 2012).

Our first hypothesis was supported by the result that germination percentages and rates were not significantly correlated with the three seed reserves in dry seeds (*P* > 0.05). Interspecific studies on the correlation between the three reserves and germination are lacking, limiting the comparison of our results with others, except that Soriano et al. (2011) found a positive relationship between germination rate and seed nitrogen content for 19 tree species from a tropical deciduous forest in Mexico. However, germination rate in our study was positively and significantly related to the soluble sugar and protein content at different germination stages (*P* < 0.05, **Table 2** and Supplementary Table S4). Soluble sugar is the main direct substance required during germination (Jennifer et al., 2002). The soluble sugar content gradually increased during germination of the six study species, which is consistent with the results from most studies (Ahmad et al., 2010; Lopes et al., 2013; Erbaş et al., 2016; Yang et al., 2016). On the other hand, the soluble sugar content was positively correlated with starch content but negatively correlated with fat content in dry seeds (*P* < 0.05). This means that soluble sugar may have mainly come from, but was not limited to, starch degradation during germination for quick energy for the embryo (Kwangho, 2005; Lopes et al., 2013). Further, the starch content was negatively related to the fat content in dry seeds of the six species (*r* = -0.71, *P* = 0.11), which may partially be explained by the negative correlation between fat content and soluble sugar.

Contrary to our second hypothesis, starch was used as the energy source mainly during imbibition for the six species (**Figure 4** and Supplementary Table S3, except *C. virgata*). The percentage of starch reduction during germination varied with species. From the dry seed (stage 0) to highest germination (stage 4), the starch content of the starchy seeds of *C. virgata* decreased only 10.1%, while that of the fatty seeds of *D. moldavica* and *L. artemisia* decreased 73.7 and 89.7%, respectively, and that of the high-protein seeds of *L. hedysaroides*, *K. scoparia*, and *A. adsurgens* decreased 52.9–78.7%. Thus, the fatty seeds may utilize more starch during germination than the starchy seeds. The previous studies have shown similar results. For example, the starch level in the high-starch seeds of *Sorghum bicolor* was reduced only 13.5% after 1–3 days from sowing (Yang et al., 2016). The total starch of four legumes (*Vigna unguiculata*, *Canavalia ensiformis*, *Stizolobium niveum*, *Lablab purpureus*) decreased 11.8–35.2% during germination (Benítez et al., 2013). The fatty seeds of *Aniba rosaeodora* showed a 29.5% reduction in starch level during germination (Lima et al., 2008). The carbohydrates level decreased 75% during germination of *Jatropha curcas* seeds, which have 40% lipids (Lopes et al., 2013).

Soluble protein was utilized for a large proportion (39.1–93.9%) from imbibition (stage 1) to the highest germination (stage 4) and even the early seedling stage (**Figure 5**), irrespective of the relative percentage of starch, protein, and fat in dry seeds. Starch is degraded by hydrolysis to sugars (Levin, 1974), and protein is degraded by the proteases and converted to polypeptide and amino acids during germination (Palmiano and Juliano, 1972). These hydrolysis products may meet the energy needs of seeds for germination (Bewley and Black, 1994; Wahid and Bounoua, 2013). The fat content remained relatively constant during germination for the six species. This study considered seed reserve degradation and utilization for the energy needs for germination. However, some components of seed reserves may promote the transition from the quiescent to the active state during germination (Catusse et al., 2008), which is certainly important and not amount dependent.

Compared with the allied crop species, we found a new result that fat in seeds of the six wild species seemed to be less important than in seeds of crop species as energy resources during germination. The fat content remained constant for the six study species (**Figure 4**). The lipid concentration in seeds of the woody legume *Dalbergia nigra* remained constant or increased slightly during germination (Ataíde et al., 2013). However, lipid content decreased 34.3 and 36.5% during seed germination of *Pisum sativum* (El-Adawy et al., 2003) and *Sorghum bicolor* (Elmaki et al., 1999), respectively. Some crop species had a great decrease of fat content during germination, e.g., 70% decrease for *A. sativa* and *L. angustifolius* (Leonova et al., 2010; Rumiyati et al., 2012). Components of fat in seeds of wild species may have other roles such as controlling the developmental switch from dormancy to germination (Footitt et al., 2002), assisting seed defense and dispersal (Finkelstein and Grubb, 2002) and fueling seedling growth after germination (Pinfield-Wells et al., 2005).

To our knowledge, this is the first study to investigate the dynamics of the three main seed reserves during germination of wild grassland species with different relative proportions of starch, protein, and fat in the seeds. We found different patterns of reserve mobilization during seed germination of wild species compared to those in domesticated crop species.

## Author Contributions

HZ and HY conceived and designed the work. MZ and LQ performed the experiments. MZ and HZ analyzed the data and wrote the paper. HZ, HY, and CB revised the manuscript.

## Conflict of Interest Statement

The authors declare that the research was conducted in the absence of any commercial or financial relationships that could be construed as a potential conflict of interest.
