# Supplementary material for: Mobilization and Role of Starch, Protein, and Fat Reserves during Seed Germination of Six Wild Grassland Species
Source: Front Plant Sci. 2018 Feb 27;9:234. doi: 10.3389/fpls.2018.00234 (PMC5835038; doi:10.3389/fpls.2018.00234)
Supplement: Supplementary file 1 [file Table_1.PDF]

TABLE S1 The sampling time (h) from seed sowing of water absorption measurements for the six species.

**Sampling**	***C. virgata***	***K. scoparia***	***L. hedysaroides***	***A. adsurgens***	***L. artemisia***	***D. moldavica***
1	2	1/3	4	4	7	7
2	4	2/3	8	8	14	14
3	6	1	12	12	21	21
4	8	4/3	16	16	28	28
5	10	5/3	20	20	35	35
6		2	24	24	42	42
