# Supplementary material for: Mobilization and Role of Starch, Protein, and Fat Reserves during Seed Germination of Six Wild Grassland Species
Source: Front Plant Sci. 2018 Feb 27;9:234. doi: 10.3389/fpls.2018.00234 (PMC5835038; doi:10.3389/fpls.2018.00234)
Supplement: Supplementary file 2 [file Table_2.PDF]

TABLE S2 The sampling time (h) from sowing to different stages of seed germination for the six species.

**Measurement stage**	***C. virgata***	***K. scoparia***	***L. hedysaroides***	***A. adsurgens***	***L. artemisia***	***D. moldavica***
Imbibition<br>Stage 1	5	1	12	12	22	22
1% germination<br>Stage 2	10	2	24	24	44	44
50% germination<br>Stage 3	14	3	48	60	66	78
Highest germination<br>Stage 4	20	10	108	84	132	132
Early seedling<br>Stage 5	44	34	132	108	156	156
