# Supplementary material for: Mobilization and Role of Starch, Protein, and Fat Reserves during Seed Germination of Six Wild Grassland Species
Source: Front Plant Sci. 2018 Feb 27;9:234. doi: 10.3389/fpls.2018.00234 (PMC5835038; doi:10.3389/fpls.2018.00234)
Supplement: Supplementary file 3 [file Table_3.PDF]

TABLE S3. Starch, soluble protein, fat and soluble sugar content (mg/g) of six species at different sampling stages during seed germination.

Reserve	Species	Dry seeds	Imbibition Stage 1	1%germination Stage 2	50%germination Stage 3	Highest germination Stage 4	Early seedling Stage 5
Starch	*C. virgata*	621.95 ±11.58^{b}	765.25 ±76.17^{b}	766.72 ±66.31^{b}	615.59 ±54.58^{b}	558.64 ±70.15^{b}	198.87 ±0.00^{a}
	K. scoparia	473.49 ±17.55^{b}	125.03 ±32.69^{a}	243.43 ±68.95^{a}	282.15 ±31.31^{ab}	185.15 ±54.22^{a}	184.22 ±65.00^{a}
	L. hedysaroides	481.88 ±63.32^{c}	425.53 ±55.34^{bc}	252.83 ±12.78^{ab}	251.94 ±16.09^{ab}	226.74 ±13.52^{ab}	174.29 ±3.50^{a}
	A. adsurgens	478.36 ±113.24^{b}	91.73 ±10.28^{a}	101.07 ±12.09^{a}	123.45 ±5.87^{a}	102.06 ±4.56^{a}	117.34 ±5.29^{a}
	L. artemisia	351.82 ±18.80^{c}	68.12 ±10.16^{b}	50.59 ±2.12^{ab}	48.48 ±4.36^{ab}	36.12 ±6.50^{a}	32.33 ±3.96^{a}
	D. moldavica	256.46 ±27.91^{b}	76.43 ±10.74^{a}	75.34 ±3.42^{a}	73.69 ±1.17^{a}	67.34 ±3.01^{a}	68.65 ±4.83^{a}
Soluble protein	*C. virgata*		0.19 ±0.07^{bc}	0.23 ±0.03^{c}	0.24 ±0.01^{c}	0.11 ±0.02^{ab}	0.03 ±0.01^{a}
	K. scoparia		3.17 ±0.53^{c}	2.92 ±0.20^{c}	2.83 ±0.20^{bc}	1.93 ±0.22^{b}	0.71 ±0.08^{a}
	L. hedysaroides		12.99 ±2.67^{b}	2.14 ±0.26^{a}	1.12 ±0.05^{a}	1.17 ±0.12^{a}	0.87 ±0.13^{a}
	A. adsurgens		0.95 ±0.16^{c}	0.93 ±0.09^{c}	0.62 ±0.02^{b}	0.39 ±0.01^{ab}	0.34 ±0.04^{a}
	L. artemisia		1.15 ±0.11^{c}	0.79 ±0.05^{b}	0.12 ±0.00^{a}	0.07 ±0.00^{a}	0.05 ±0.04^{a}
	D. moldavica		0.32 ±0.04^{a}	0.31 ±0.04^{a}	0.27 ±0.13^{a}	0.20 ±0.04^{a}	0.14 ±0.03^{a}
Fat	*C. virgata*	62.16 ±4.07^{a}	71.76 ±3.65^{a}	70.96 ±3.29^{a}	71.23 ±5.87^{a}	67.64 ±3.98^{a}	66.41 ±3.53^{a}
	K. scoparia	112.32 ±18.20^{a}	142.61 ±10.40^{a}	134.53 ±0.49^{a}	142.24 ±2.71^{a}	127.77 ±1.60^{a}	111.33 ±13.65^{a}
	L. hedysaroides	86.11 ±7.86^{a}	112.86 ±4.48^{bc}	116.78 ±5.86^{c}	103.88 ±5.43^{bc}	99.29 ±6.16^{ab}	96.10 ±1.63^{ab}
	A. adsurgens	94.00 ±10.37^{c}	74.34 ±2.68^{ab}	77.97 ±2.80^{abc}	79.83 ±4.59^{abc}	88.52 ±5.89^{bc}	71.18 ±2.72^{a}
	L. artemisia	358.23 ±2.64^{a}	352.79 ±6.38^{a}	341.50 ±5.07^{a}	348.12 ±15.62^{a}	347.80 ±8.96^{a}	334.01 ±11.21^{a}
	D. moldavica	200.40 ±2.37^{a}	266.57 ±3.32^{c}	239.80 ±9.40^{b}	204.50 ±14.47^{a}	210.40 ±5.09^{a}	197.67 ±9.08^{a}
Soluble sugar	*C. virgata*		225.86 ±12.50^{a}	245.63 ±6.11^{a}	235.05 ±12.95^{a}	359.14 ±12.21^{b}	393.37 ±39.15^{b}
	K. scoparia		183.51 ±5.75^{a}	202.73 ±8.67^{ab}	222.17 ±16.16^{ab}	224.26 ±13.58^{b}	238.58 ±16.37^{b}
	L. hedysaroides		53.75 ±12.24^{a}	68.57 ±6.66^{ab}	93.64 ±2.28^{bc}	103.34 ±6.54^{c}	107.36 ±9.77^{c}
	A. adsurgens		126.99 ±11.31^{bc}	129.06 ±8.97^{bc}	146.60 ±2.54^{c}	112.25 ±1.36^{ab}	105.58 ±3.75^{a}
	L. artemisia		15.81 ±2.37^{a}	17.51 ±0.85^{a}	23.22 ±1.47^{b}	25.19 ±1.96^{b}	28.16 ±1.02^{b}
	D. moldavica		28.98 ±4.14^{a}	31.13 ±0.42^{a}	32.10 ±2.13^{a}	30.35 ±0.54^{a}	32.95 ±2.42^{a}

Different small letters indicate significant difference at $P < 0.05$ level.
