# Supplementary material for: Mobilization and Role of Starch, Protein, and Fat Reserves during Seed Germination of Six Wild Grassland Species
Source: Front Plant Sci. 2018 Feb 27;9:234. doi: 10.3389/fpls.2018.00234 (PMC5835038; doi:10.3389/fpls.2018.00234)
Supplement: Supplementary file 4 [file Table_4.PDF]

TABLE S4 Correlation coefficients between seed reserves in dry seeds, germination percentage (GP), germination rate (GR) and soluble sugar, soluble protein contents at different germination stages.

**Source of Variation**	**Starch**	**Protein**	**Fat**	**GP**	**GR**
1-Soluble sugar	0.68**	0.36	-0.67**	-0.11	0.58*
2- Soluble sugar	0.61**	0.35	-0.67**	-0.06	0.60**
3- Soluble sugar	0.63**	0.47	-0.70**	-0.02	0.65**
4- Soluble sugar	0.64**	0.22	-0.62**	-0.02	0.48*
5- Soluble sugar	0.65**	0.16	-0.57*	0.06	0.47*
1-Soluble protein	0.25	0.43	-0.27	0.21	-0.04
2-Soluble protein	0.06	0.73**	-0.28	0.37	0.70**
3-Soluble protein	0.15	0.66**	-0.35	0.31	0.90**
4-Soluble protein	0.16	0.67**	-0.40	0.30	0.77**
5-Soluble protein	0.21	0.67**	-0.42	0.26	0.40

Germination stages: 1, imbibition; 2, 1% germination; 3, 50% germination; 4, highest germination; 5, early seedling
